# Myopia control efficacy of spectacle lenses with highly aspherical lenslets: results of a 5-year follow-up study

**DOI:** 10.1186/s40662-025-00427-3

**Published:** 2025-03-05

**Authors:** Xue Li, Yingying Huang, Chenyao Liu, Xindan Chang, Zaifeng Cui, Qiulin Yang, Björn Drobe, Mark A. Bullimore, Hao Chen, Jinhua Bao

**Affiliations:** 1 National Clinical Research Center for Ophthalmology and Optometry, Eye Hospital, Wenzhou Medical University, 270 Xueyuan Road, Wenzhou, 325027 Zhejiang China; 2 R&D, EssilorLuxottica, Singapore, Singapore; 3 College of Optometry, University of Houston, Houston, TX USA

**Keywords:** Myopia, Axial length, Aspherical lenslets, Extrapolated single-vision spectacle lenses group

## Abstract

**Purpose:**

To evaluate myopia control efficacy in myopic children wearing spectacle lenses with highly aspherical lenslets (HAL) for 5 years.

**Methods:**

This is a randomized, double-masked extended trial. Myopic children aged 8 to 13 years who were originally allocated to the HAL group in the 2-year clinical trial. The HAL group underwent a 5-year assessment for myopia progression using cycloplegic spherical equivalent refraction (SER) and axial length (AL). An extrapolated single-vision spectacle lenses (ESVL) group was used as a control group. The 5-year myopia progression and axial elongation of the ESVL group was calculated based on the 2-year data from the single-vision spectacle lenses group in the same clinical trial, and the data for the following 3 years was estimated by assuming an annual reduction in SER by 9.7% and in AL by 15%. A generalized linear model approach was used to evaluate the treatment efficacy. The validity of the ESVL group was evaluated by comparing myopia progression in the first year of the 3-year estimates with a single-vision spectacle lenses (SVL2) group from a 1-year extended study of the same clinical trial.

**Results:**

Forty-three participants from the original HAL group completed the 5-year visit (74%). Five-year myopia progression [mean ± standard error (SE)] in the HAL group was − 1.27 ± 0.14 D. Compared with the ESVL (− 3.03 ± 0.18 D), myopia progression was − 1.75 ± 0.24 D less for the HAL group (*P* < 0.001). The mean AL elongation over 5 years was 0.67 ± 0.06 mm for the HAL group compared with 1.40 mm in the ESVL group (*P* < 0.001), AL elongation was slower by 0.72 ± 0.10 mm for the HAL group (*P* < 0.001). No significant differences were found for myopia (− 0.58 ± 0.04 D vs*.* − 0.56 ± 0.05 D) or AL elongation (0.28 ± 0.02 mm vs. 0.28 ± 0.02 mm) between the ESVL group and SVL2 group (*P*_SER_ = 0.83; *P*_AL_ = 0.93) in year 3.

**Conclusions:**

In this 5-year study, HAL spectacles reduced the rate of myopia progression and axial elongation, preventing the equivalent of 3 years of myopia progression and axial elongation. Long-term use of HAL spectacles also decreased the incidence of high myopia. Extrapolated control groups are valid for evaluating myopia progression in long-term studies.

*Trial registration* The study was registered at the Chinese Clinical Trial Registry (ChiCTR2100047262), https://www.chictr.org.cn/showproj.html?proj=127182.

**Supplementary Information:**

The online version contains supplementary material available at 10.1186/s40662-025-00427-3.

## Background

Myopia has significantly increased in prevalence worldwide in recent decades [1]. Myopia can have a major adverse impact, potentially leading to irreversible visual impairment and imposing heavy economic burdens [2–4]. It has been predicted that almost half of the world’s population will be myopic by 2050, with 10% experiencing high myopia [5]. Consequently, slowing myopia progression (myopia control) has gained increased attention [6].

Studies of various interventions for myopia control, including optical and pharmacological approaches [7–12] have shown significant efficacy. Nonetheless, there are still some concerns to address when applying them in clinical practice, such as the long-term efficacy and safety of new technologies [13, 14]. Since myopia usually develops during elementary school [15, 16] and progresses until much later [17], intervention duration of 5 years or more may be necessary for many myopic children. Unfortunately, most studies of myopia control efficacy have employed a study duration of 2 years or less, due in part to the ethics of withholding treatment [18]. The short-term efficacy of initial treatment may fail to adequately reflect long-term treatment outcomes [7, 11, 19]. Therefore, further investigation into the long-term myopia control efficacy of interventions is needed.

Spectacle lenses with highly aspherical lenslets (HAL), generate a myopia control signal volume in front of the retina. In a 2-year clinical trial, the use of HAL has been shown to slow myopia progression and axial length (AL) elongation by 0.80 D and 0.35 mm, respectively [12]. This study aimed to determine the longer-term myopia control efficacy of HAL over 5 years, by comparing it with an extrapolated single-vision spectacle lenses (ESVL) group.

## Methods

### Study design

Originally in 2018, 170 children, aged 8 to 13 years, were recruited and randomly assigned to either single-vision spectacle lenses (SVL), HAL (n = 58), or spectacle lenses with slightly aspherical lenslets (SAL) for a 2-year randomized control trial (RCT) [12, 20]. After completing the RCT, 52 children wearing HAL participated in a 1-year extended study (year 3), and a new single-vision control group (SVL2 group) was recruited to evaluate myopia progression in the third year [21]. Thereafter, participants who had worn HAL continuously for 3 years were invited to join a new 2-year extension follow-up study (years 4–5, ChiCTR2100047262) representing a total of 5 years of treatment with HAL. The Ethics Committee of the Eye Hospital of Wenzhou Medical University approved this study (2021-087-K-74), and all procedures were carried out following the tenets of the Declaration of Helsinki. Written informed consent and assent were obtained from the participants and their parents or guardians after verbal and written explanations of the objectives and consequences of the study were provided.

### ESVL group

The extrapolations of the myopia changes in spherical equivalent refraction (SER) and AL over 5 years in the ESVL group were based on the initial 2 years of data from the single-vision spectacle lenses (SVL) group in the same clinical trial. For the following 3 years, an average annual decrease in SER progression of 9.7% [22] and a 15% reduction in AL elongation were estimated [23]. Smotherman et al. used 127 evaluations with a multivariate linear mixed-effects meta-analysis model to assess refractive error change, finding a 9.7% annual decrease (95% CI: 5.4% to 13.9%, *P* < 0.0001) [22]. Brennan et al. analyzed 78 studies, including longitudinal and retrospective studies, along with control subjects from randomized clinical trials, to evaluate the axial elongation rate using a multivariate linear mixed-effects meta-regression model. Their findings indicated that both Asian children and non-Asians exhibited a 15% annual decline in axial elongation as age increased (95% CI: 12% to 17%, *P* < 0.0001) [23]. AL and SER progression were extrapolated for individual children in order to estimate the proportion that would have progressed to high myopia (− 6.00 D or worse). The validity of the ESVL group was assessed by comparing changes in myopia progression and AL elongation in the first year of the 3-year extrapolation with the SVL2 group of 48 children from a 1-year extended study of this RCT.

### Study procedures

The data measurement procedures followed those used in the 3-year follow-up study [21]. SER and AL were measured annually in the 4th and 5th years. SER was obtained from cycloplegic autorefraction using an autorefractor (KR-800, TOPCON Corporation, Japan). For cycloplegia, two drops of 0.5% tropicamide and 5% phenylephrine eyedrops were administered 5 min apart, and refraction was performed at least 30 min after the last drop. AL was measured by an optical low-coherence reflectometry device (Lenstar 900, Haag-Streit AG Switzerland). The average values of ten autorefractions and five AL measurements were used for data analysis.

### Statistical analysis

Statistical analyses were performed using SPSS 25.0. The baseline characteristics and changes in SER and AL are presented as the mean ± standard error (SE). Data from the right eye were used for the analysis due to a high correlation between both eyes over 5 years in SER (r = 0.77, *P* < 0.001) and AL (r = 0.85, *P* < 0.001). Kolmogorov–Smirnov tests were performed to check for distribution, and unpaired t-tests and Mann–Whitney U tests were used as appropriate. Repeated-measures ANOVA with post hoc LSD test were used to assess intergroup differences. A generalized linear model approach, with adjustments for confounding covariates, including baseline age, sex, SER, AL, and age at myopia onset, was used to evaluate the treatment efficacy. Two-sided *P* values of less than 0.05 were considered statistically significant.

## Results

### Participants

Of the 51 participants who had worn HAL for 3 years, 44 continued for the follow-up study into years 4 and 5 (86%). Eventually, 43 children completed this 2-year extended study (Table 1). One child was excluded for missing the 48-month follow-up and replacing her spectacles outside the study. The ESVL group included 50 participants from the same clinical trial who had worn SVL for 2 years (Table 1). The flowchart of this study is shown in Figure S1.

**Table 1:** Baseline demographics and ocular characteristics of participants in the HAL and ESVL groups

Parameter	Mean (SE)		*P* value (*t*-test / Chi-squared test)
	HAL (n = 43)	ESVL (n = 50)	
Age (years)	10.7 (0.2) [10.4, 11.1]	10.4 (0.2) [10.0, 10.7]	0.15
Sex Female, No. (%) Male, No. (%)	23 (53.5) 20 (46.5)	21 (42.0) 29 (58.0)	0.27
SER (D)	− 2.81 (0.16) [− 3.13, − 2.48]	− 2.44 (0.12) [− 2.69, − 2.19]	0.07
Axial length (mm)	24.82 (0.11) [24.60, 25.04]	24.77 (0.09) [24.58, 24.96]	0.74
Age at myopia onset (years)	9.3 (0.2)	9.3 (0.2)	0.96
	[8.9, 9.8]	[8.9, 9.8]	
Parents with myopia, No. (%)			0.50
0	15 (34.9)	12 (24.0)	
1	14 (32.6)	18 (36.0)	
2	14 (32.6)	20 (40.0)	

*HAL* = spectacle lenses with highly aspherical lenslets; *ESVL* = extrapolated single-vision spectacle lenses; *SE* = standard error; *SER* = spherical equivalent refraction; *D* = diopters

### ESVL group

**Fig. 1:**
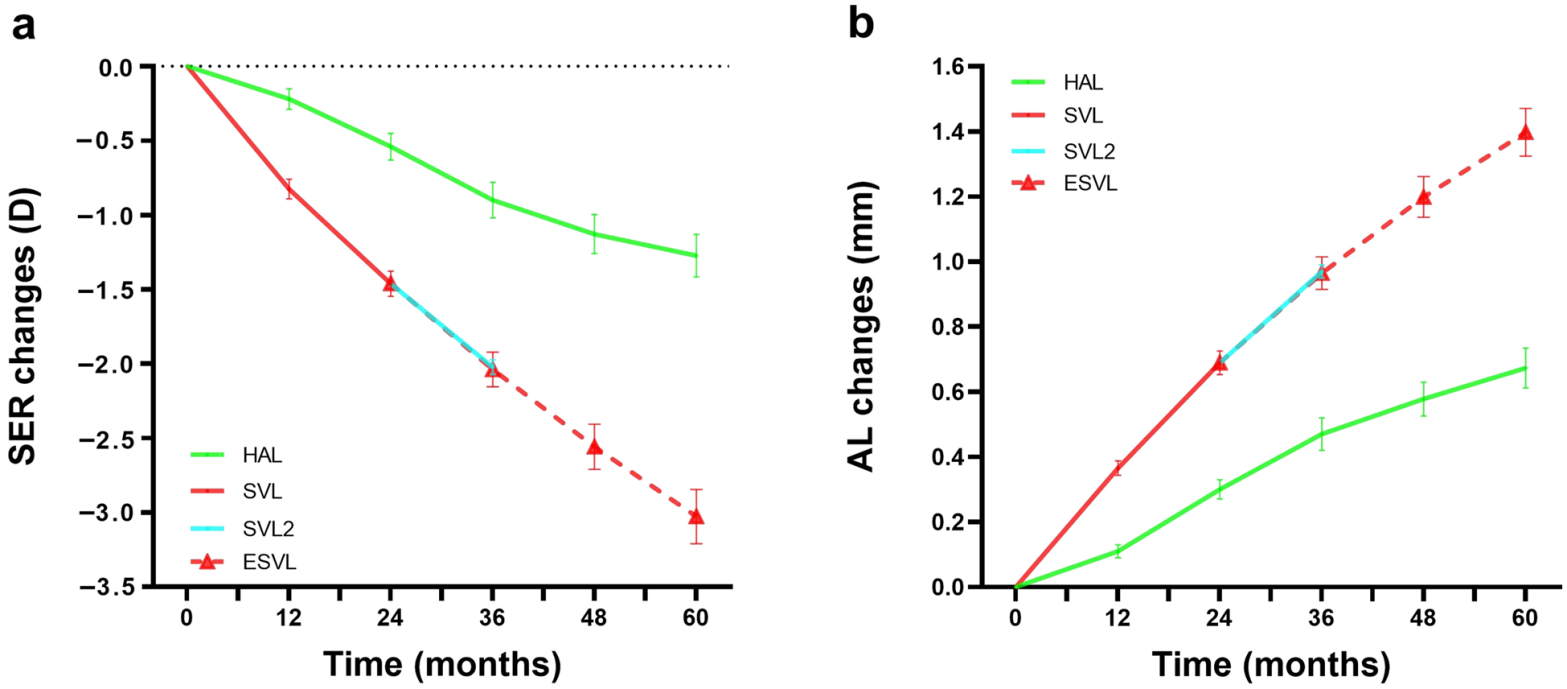
The unadjusted changes in spherical equivalent refraction (SER) and axial length (AL) over the 5-year study for HAL and ESVL groups. **a** SER changes; **b** AL changes. Error bars represent standard errors of the mean. HAL, spectacle lenses with highly aspherical lenslets; SVL, single-vision spectacle lenses (data from the 2-year clinical trial); SVL2, single-vision spectacle lenses (data from a 1-year extended study of the same clinical trial); ESVL, extrapolated single-vision spectacle lenses (data was calculated based on the 2-year data from the SVL group in the same clinical trial, and the data for the following 3 years was estimated by assuming an annual reduction in SER by 9.7% [22] and in AL by 15% [23]

﻿In the ESVL group, the 5-year mean ± SE myopia progression and AL elongation were − 3.03 ± 0.18 D and − 1.40 ± 0.07 mm, respectively (Table 2 and Fig. 1). During the 3rd year, the changes in SER (− 0.58 ± 0.04 D vs. − 0.56 ± 0.05 D, t =  − 0.22, *P* = 0.83) and AL (0.28 ± 0.02 mm vs. 0.28 ± 0.02) mm, t =  − 0.90, *P* = 0.93) were comparable to those of the SVL2 control group (Table 2). There were no significant differences in age, sex, baseline SER, baseline AL, and the number of myopic parents between the ESVL and SVL2 groups (all *P* ≥ 0.16, Table S1).

**Table 2:** Mean (SE) and 95% confidence interval of the SER and AL from baseline to 60 months in HAL, ESVL, and historical SVL2 groups

Time	HAL	SVL/ ESVL	SVL2	HAL	SVL/ ESVL	SVL2	HAL	SVL/ ESVL	SVL2
(months)	(n = 43)	(n = 50)	(n = 48)	(n = 43)	(n = 50)	(n = 48)	(n = 43)	(n = 50)	(n = 48)
	SER (D)			Myopia progression (D)			Myopia progression in each year (D)		
0	− 2.81 (0.16)	− 2.44 (0.12)	–	–	–	–	–	–	–
	[− 3.13, − 2.48]	[− 2.69, − 2.12]							
12	− 3.02 (0.16)	− 3.27 (0.12)	–	− 0.22 (0.07)	− 0.83 (0.07)	–	− 0.22 (0.07)	− 0.83 (0.07)	–
	[− 3.34, − 2.70]	[− 3.51, − 3.02]		[− 0.35, − 0.08]	[− 0.96, − 0.69]		[− 0.35, − 0.08]	[− 0.96, − 0.69]	
24	− 3.35 (0.18)	− 3.90 (0.13)	− 3.73 (0.14)	− 0.54 (0.09)	− 1.46 (0.08)	–	− 0.33 (0.06)	− 0.64 (0.05)	–
	[− 3.71, − 2.99]	[− 4.16, − 3.65]	[− 4.02, − 3.44]	[− 0.73, − 0.36]	[− 1.63, − 1.29]		[− 0.44, − 0.22]	[− 0.73, − 0.54]	
36	− 3.70 (0.19)	** − 4.49 (0.14)**	− 4.29 (0.15)	− 0.90 (0.12)	** − 2.04 (0.12)**	− 0.56 (0.05)	− 0.35 (0.06)	** − 0.57 (0.04)**	− 0.56 (0.05)
	[− 4.08, − 3.32]	**[− 4.77, 4.19]**	[− 4.58, − 3.99]	[− 1.13, − 0.66]	**[− 2.27, − 1.80]**	[− 0.67, − 0.45]	[− 0.46, − 0.24]	**[− 0.66, − 0.49]**	[− 0.67, − 0.45]
48	− 3.93 (0.20)	** − 5.00 (0.17)**	–	− 1.13 (0.13)	** − 2.56 (0.15)**	–	− 0.23 (0.04)	** − 0.52 (0.04)**	–
	[− 4.34, − 3.52]	**[− 5.33, − 4.66]**		[− 1.39, − 0.86]	**[− 2.86, − 2.26]**		[− 0.31, − 0.16]	**[− 0.60, − 0.44]**	
60	− 4.01 (0.22)	** − 5.47 (0.19)**	–	− 1.27 (0.14)	** − 3.03 (0.18)**	–	− 0.15 (0.04)	** − 0.47 (0.04)**	–
	[− 4.51, − 3.64]	**[− 5.86, − 5.08]**		[− 1.56, − 0.99]	**[− 3.40, − 2.66]**		[− 0.23, − 0.07]	**[− 0.54, − 0.40]**	

Time	AL (mm)			AL elongation (mm)			AL elongation in each year (mm)		
(months)									
0	24.82 (0.11)	24.77 (0.09)	–	–	–	–	–	–	–
	[24.60, 25.04]	[24.58, 24.96]							
12	24.93 (0.12)	25.14 (0.19)	–	0.11 (0.02)	0.37 (0.02)	–	0.11 (0.02)	0.37 (0.02)	–
	[24.69, 25.16]	[24.95, 25.32]		[0.06, 0.16]	[0.32, 0.41]		[0.06, 0.16]	[0.32, 0.41]	
24	25.12 (0.13)	25.46 (0.09)	25.47 (0.12)	0.30 (0.04)	0.69 (0.04)	–	0.19 (0.02)	0.32 (0.02)	–
	[24.86, 25.37]	[25.27, 25.65]	[25.24, 25.71]	[0.22, 0.37]	[0.62, 0.76]		[0.15, 0.23]	[0.29, 0.36]	
36	25.28 (0.13)	**25.74 (0.10)**	25.75 (0.12)	0.47 (0.05)	**0.96 (0.05)**	0.28 (0.02)	0.17 (0.02)	**0.28 (0.02)**	0.28 (0.02)
	[25.03, 25.54]	**[25.54, 25.93]**	[25.52, 25.99]	[0.37, 0.56]	**[0.86, 1.07]**	[0.25, 0.31]	[0.13, 0.20]	**[0.24, 0.31]**	[0.25, 0.31]
48	25.40 (0.13)	**25.97 (0.10)**	–	0.58 (0.05)	**1.20 (0.06)**	–	0.11 (0.01)	**0.23 (0.01)**	–
	[25.14, 25.66]	**[25.76, 26.18]**		[0.47, 0.68]	**[1.07, 1.32]**		[0.08, 0.14]	**[0.21, 0.26]**	
60	25.49 (0.13)	**26.17 (0.11)**	–	0.67 (0.06)	**1.40 (0.07)**	–	0.10 (0.01)	**0.20 (0.01)**	–
	[25.23, 25.76]	**[25.95, 26.39]**		[0.55, 0.80]	**[1.25, 1.55]**		[0.07, 0.12]	**[0.18, 0.22]**	

*SE* = standard error; *SER* = spherical equivalent refraction; *AL* = axial length; *HA*L = spectacle lenses with highly aspherical lenslets; *ESVL* = the extrapolated single-vision spectacle lenses group proposed by Brennan et al. [23] and Smotherman et al. [22] in month 36–60; *SVL *= single-vision spectacle lenses as a control in month 0–24; *SVL2* = new single-vision spectacle lenses group as a control in months 24–36; The blocks in bold indicate the 36–60 months of the ESVL group

### Changes in SER over 5 years of HAL wear

The HAL group exhibited a mean change in SER of − 1.27 ± 0.14 D over 5 years (Table 2 and Fig. 1). There was a significant change in myopia progression over time (F_4, 0.31_ = 2.88, *P* = 0.02), with the fastest myopia progression occurring in the 3rd year and the slowest in the 5th year (*P* = 0.02, Table 2). The 5-year myopia progression in the HAL group was significantly less than that in the ESVL group (mean difference, 1.75 ± 0.24 D, t = 7.39, *P* < 0.001).

In the generalized linear model analysis, the lower baseline age (95% CI: 0.28 to 0.82, *P* < 0.001) was significantly associated with faster myopia progression. After adjusting for baseline age, sex, SER, AL, and age at myopia onset, the model-adjusted mean ± SE changes in SER were − 1.38 ± 0.15 D and − 2.94 ± 0.14 D for the HAL and ESVL groups, respectively. Compared to the ESVL group, the adjusted difference in mean ± SE SER was 1.56 D lower (95% CI: 1.14 to1.99, *P* < 0.001) in the HAL group.

### Changes in AL over 5 years of HAL wear

The HAL group demonstrated a mean AL elongation of 0.67 ± 0.06 mm over 5 years (Table 2 and Fig. 1), significantly less than that in the ESVL group (mean difference, 0.72 ± 0.10 mm, t =  − 7.45, *P* < 0.001). The change in AL elongation over time was statistically significant (F_3.48, 0.08_ = 6.61, *P* < 0.001). Post hoc analyses revealed that the rate of AL elongation was fastest in the 2nd and 3rd year and slowest in the 5th year (all *P* ≤ 0.001, Table 2).

In the generalized linear model analysis, lower baseline age (95% CI: − 0.41 to − 0.22, *P* < 0.001), earlier myopia onset (95% CI: 0.005 to 0.15, *P* = 0.04), and higher baseline AL (95% CI: 0.05 to 0.31, *P* = 0.008) was significantly associated with faster AL elongation. After adjustment, the mean ± SE AL elongation was 0.73 ± 0.06 mm and 1.35 ± 0.05 mm for the HAL and ESVL groups, respectively. There was a significant reduction in AL elongation of 0.62 ± 0.08 mm (95% CI: 0.47 to 0.78, *P* < 0.001) in the HAL group compared to the ESVL group.

### Distribution of myopia progression in HAL and ESVL groups

**Fig. 2:**
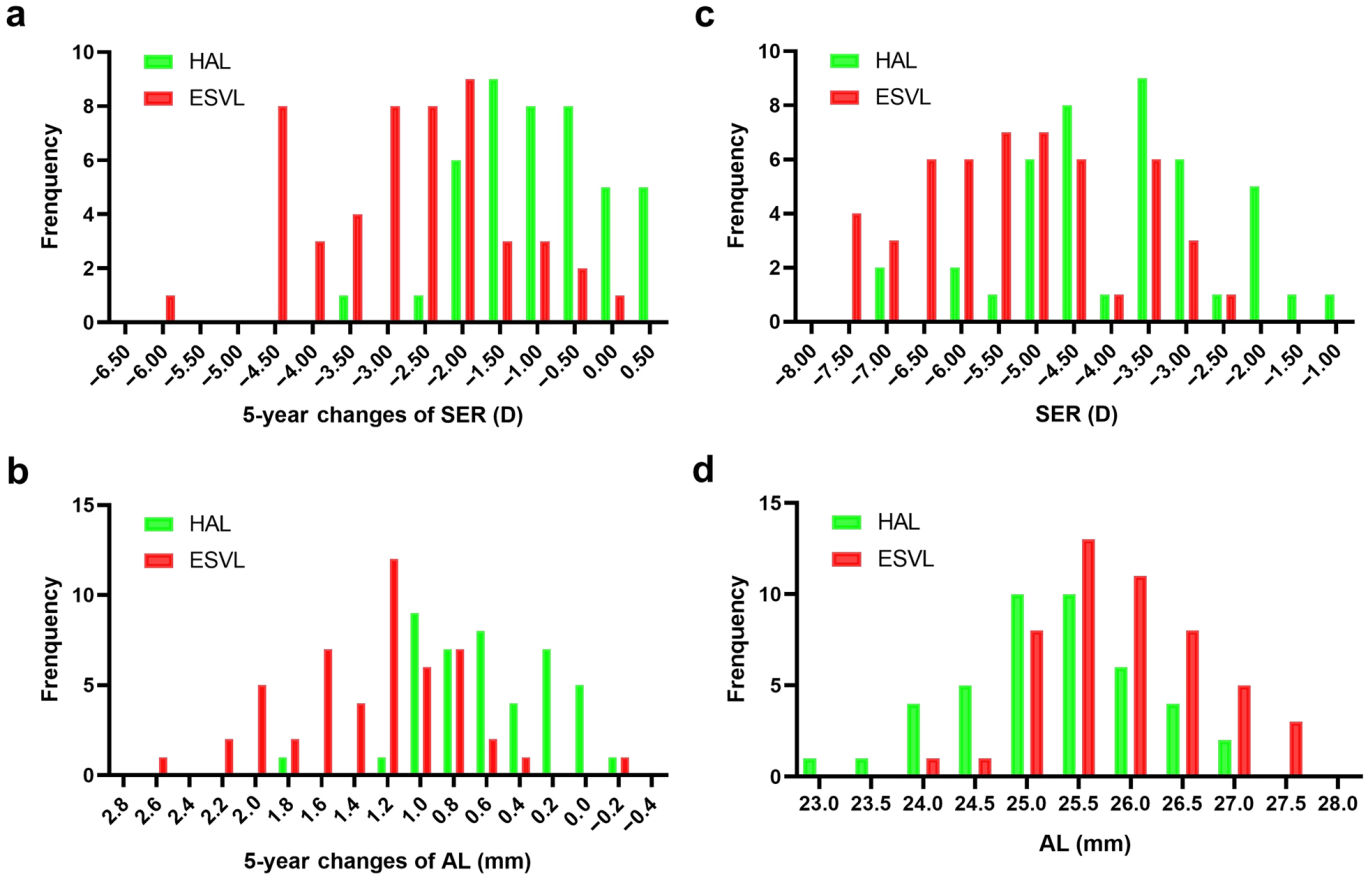
Distribution of changes in SER (**a**) and AL (**b**) of HAL and ESVL groups during 5 follow-up years, and distribution of SER (**c**) and AL (**d**) in HAL and ESVL groups after 5 years. AL, axial length; SER, spherical equivalent refraction; HAL, spectacle lenses with highly aspherical lenslets; ESVL, extrapolated single-vision spectacle lenses

In the HAL group, 5% of participants experienced myopia progression of more than 2.50 D over the 5-year period, while in the ESVL group, this progression was projected in 64% of children (F = 35.62, *P* < 0.001, Fig. 2a). Conversely, 12% of participants in the HAL group had myopia progression of 0.00 D or less, compared to 2% in the ESVL group. Furthermore, after 5 years, the incidence of high myopia (− 6.00 D or worse) was 9% in the HAL group and 38% in the ESVL group (F = 10.23, *P* = 0.002).

### Distribution of AL in HAL and ESVL groups

In the HAL group, 12% of participants experienced an AL elongation of less than 0.2 mm over 5 years, compared with 2% in the ESVL group. Conversely, 26% of the HAL group experienced an AL elongation equal to or greater than 1.00 mm (Fig. 2b) compared to a projection of 78% of the ESVL group (F = 25.78, *P* < 0.001, Fig. 2b). Moreover, after 5 years, 28% of the HAL group had an AL greater than 26.0 mm compared with a projected 54% in the ESVL groups (F = 6.46, *P* = 0.011).

### Adverse events

No complaints, such as blur, dizziness, headache and other symptoms were reported verbally during the extended study period of 4–5 years.

## Discussion

The current study demonstrates the long-term efficacy of HAL for myopia control. The 5-year myopia progression and axial elongation in the HAL group were comparable to the 2-year changes in the initial control group wearing single-vision spectacle lenses (SER, − 1.27 D vs. − 1.46 D; AL, 0.67 mm vs. 0.69 mm). Note that this comparison does not rely on any extrapolation of data in the single vision group. Similarly, compared to the extrapolated single-vision control group, wearing HAL for 5 years slowed myopia progression by 1.75 D and AL elongation by 0.72 mm. This outcome is comparable to the results of Low-concentration Atropine on Myopia Progression (LAMP) Study [24] that estimated 0.05% atropine slowed myopia progression by 1.67 D and axial elongation by 0.75 mm, also using an ESVL group. A long-term study of children wearing dual-focus soft contact lenses for 6 years estimated that axial elongation was slowed by 0.52 mm, again using an ESVL group for the last 3 years [25]. The 6-year follow-up of the 93 children originally assigned to wear defocus incorporated multiple segments (DIMS) spectacles lenses reported that only 36 of them wore the lenses for the entire 6 years (39%) and did not attempt to quantify the long term slowing of progression [26]. Finally, 5-year wear of overnight orthokeratology lenses slowed AL elongation by 0.42 mm compared to SVL [27]. Furthermore, wearing HAL for 5 years reduced the risk of progressing to high myopia (− 6.00 D or worse) from 38% to 9%, indicating that the relative risk of developing high myopia in the HAL group was four times lower than in the ESVL group.

**Fig. 3:**
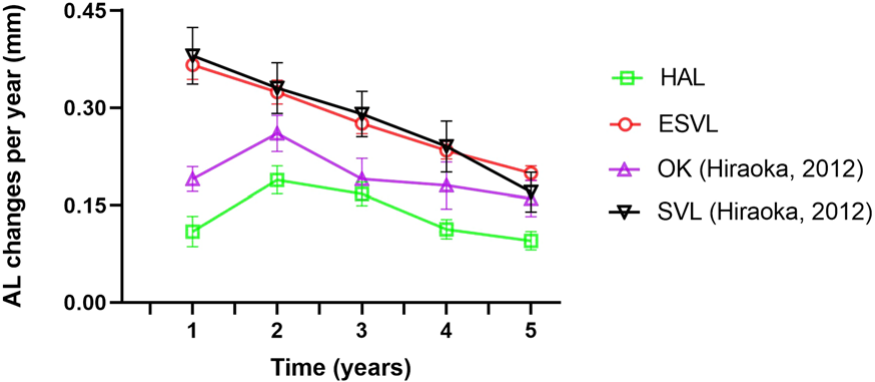
Changes in axial length (AL) elongation per year from baseline to 5 years. Error bars represent standard errors of the mean. HAL, group wearing spectacle lenses with highly aspherical lenslets in this study; ESVL, group for the extrapolated single-vision spectacle lenses group in this study; OK, group wearing orthokeratology lenses, based on a 5-year follow-up study by Hiraoka et al.[27]; SVL, group wearing single-vision spectacle lenses, based on a 5-year follow-up study by Hiraoka et al.[27]

This study developed an extrapolated single-vision control group to evaluate the long-term efficacy of myopia control. Data from the SVL group in our clinical trial was used for the first 2 years, and estimates were made for the following 3 years based on an average annual decrease in SER by 9.7% [22] and in AL by 15% [23]. This approach was proposed due to challenges in conducting placebo-controlled randomized clinical trials. Similar approaches were used in recent studies to evaluate the 3-year efficacy of low-concentration atropine [28] and the 6-year efficacy of dual-focus myopia control contact lenses [25]. To validate the accuracy of the extrapolated control group used in this study, the myopia progression in the first year of the 3-year estimates was compared with data from the SVL2 group of our extended 3rd-year study, showing comparable results (Fig. 1). Additionally, based on our findings over the previous three years, within the SVL groups, AL elongation measured 0.37 mm (year 1), 0.32 mm (year 2), and 0.28 mm (year 3), indicating an approximate 15% annual decrease. The estimated 5-year myopia progression of the extrapolated control group in this study was − 3.03 D, and based on the medial records of patients at the same Eye Hospital, it was found that the average 7-year myopia progression in myopic children (median age at the initial visit, 9 years old) wearing SVL was around − 4.00 D [29]. Additionally, the estimated 5-year AL elongation of the extrapolated control group in this study was 1.40 mm, consistent with observations 1.41 mm in the SVL group in Hiraoka et al.’s study (Fig. 3) [27]. These results confirm the plausibility and usability of using an extrapolated single-vision lenses group for evaluating myopia control efficacy. In the HAL group, myopia progression and axial elongation were the fastest during the 2nd and 3rd years. This could be attributed to the temporary lockdown and increased online learning at home during the 2nd and 3rd years (July 2019 to September 2021) when the COVID-19 pandemic [30, 31].

The fastest progression would also be anticipated in the 2nd and 3rd years due to the reduced efficacy following the first year [18]. Additionally, the subsequent decrease in myopia progression could be due to aging. Age was found to be an important factor associated with myopia progression and axial elongation [32, 33]. The axial elongation was high between the ages of 8 and 11 and significantly lower between the ages of 12 and 16 [33]. This study revealed that axial elongation was associated with baseline age, consistent with previous studies indicating that younger baseline age is associated with faster myopia [23, 32, 34–37].

This study found that 95% of the children who wore HAL experienced myopia progression of less than 2.50 D over 5 years. In contrast, only 36% of the ESVL group showed similar progression (Fig. 2a). Additionally, 12% of the participants in the HAL group experienced no increase in myopia over the 5-year period. After 5 years, the study noted that 9% of the children in the HAL group had high myopia (− 6.00 D or worse), compared to 38% in the ESVL group. In a retrospective study of myopic children (6–10 years) wearing SVL over an average 7 years, it was found that children with SER between − 2.00 D and − 4.00 D had 67% risk of developing high myopia, and children with SER between − 0.50 D and − 2.00 D had 34% risk of developing high myopia at 16 years old [29]. Therefore, long-term wearing of HAL could significantly reduce the incidence of high myopia. Additionally, Fig. 2a suggests a possible bimodal distribution. Based on a subgroup analysis of baseline age, SER, and AL, the SVL subgroup, which includes 9 participants with fastest myopia progression (− 4.96 D over 5 years), was significantly younger at baseline than the others (9.6 ± 1.4 years vs. 10.5 ± 1.4 years, *P* = 0.03).

The limitation of this study was that during this new 2-year extension follow-up study (years 4–5), 7 children (14%) discontinued due to school commitments, while 44 continued to participate in the study. We analyzed the data from 51 children over 3 years and found no statistical differences in baseline age, SER, AL, and myopia progression or AL elongation between the children who continued with the follow-up at years 4 and 5 and those who discontinued (all *P* < 0.05, Table S2), and their differences in myopia progression and axial elongation were less than 0.20 D and 0.07 mm per year, respectively. Moreover, we did not track wearing time in years 4 and 5. In our earlier study, participants reported an average daily wearing time of 12.9 h during the first year [20], which increased to 13.4 h in year 2 [12] and 15.2 h in year 3 [21]. Therefore, we work under the assumption that the participants had become long-term habitual users of HAL. The consistent slower progression in years 4 and 5 compared to the ESVL group suggests that this assumption is valid.

## Conclusion

In Chinese children suffering from myopia, wearing HAL spectacles effectively reduced the rate of myopia progression and eye growth over 5 years when compared with a ESVL group. Long-term use of HAL lenses decreased the incidence of high myopia. The children wearing HAL lenses will be monitored for another 2 years, after their initial 5 years of use.

## Supplementary Information

Additional file 1.

## Data Availability

The data that support the findings of this study are available from the corresponding author upon reasonable request.

## Abbreviations

AL: Axial length
D: Diopters
HAL: Spectacle lenses with highly aspherical lenslets
ESVL: Extrapolated single-vision spectacle lenses
