# Supplementary material for: Myopia control efficacy of spectacle lenses with highly aspherical lenslets: results of a 5-year follow-up study
Source: Eye Vis (Lond). 2025 Mar 5;12:10. doi: 10.1186/s40662-025-00427-3 (PMC11881363; doi:10.1186/s40662-025-00427-3)

**Additional files**

Table S1. Baseline demographic and ocular characteristics of participants in the SVL2 and the ESVL groups.

| Clinical value | Mean (SE) |  | *P* value  (*t*-test / Chi-square test) |
| --- | --- | --- | --- |
|  | SVL2 (n=48) | ESVL (n=50) |  |
| Age (years) | 12.0 (0.2) [11.6, 12.4] | 12.4 (0.2) [12.0, 12.7] | 0.16 |
| Sex  Female, No. (%)  Male, No. (%) | 26 (54.2) 22 (45.8) | 21 (42.0) 29 (58.0) | 0.23 |
| SER (D) | −3.73 (0.14) [−4.02, −3.44] | −3.90 (0.13) [−4.16, −3.65] | 0.36 |
| Axial length (mm) | 25.47 (0.12) [25.24, 25.71] | 25.46 (0.09) [25.27, 25.65] | 0.93 |
| Parents with myopia, No. (%) |  |  | 0.65 |
| 0 | 8 (16.7) | 12 (24.0) |  |
| 1 | 18 (37.5) | 18 (36.0) |  |
| 2 | 22 (45.8) | 20 (40.0) |  |

SVL2 = new single-vision spectacle lenses group as a control in months 24–36; ESVL = extrapolated single-vision spectacle lenses; SE = standard error; SER = spherical equivalent refraction; D = diopters

Table S2. Baseline demographic and ocular characteristics, myopia progression, and axial length elongation in 3 years of participants wearing HAL, which continued and discontinued after 3 years.

| Clinical value | Continue (n=44) | Discontinued (n=7) | *P* value (*t*-test/ Chi- square test |
| --- | --- | --- | --- |
| Age (years) | 10.73 (0.17) [10.38, 11.07] | 10.57 (0.53) [9.28, 11.86] | 0.74 |
| Sex  Female, No. (%)  Male, No. (%) | 24 (54.5) 20 (45.5) | 2 (28.6) 5 (71.4) | 0.20 |
| SER (D) | −2.82 (0.11) [−3.14, −2.50] | −2.05 (0.24) [−2.65, −1.46] | 0.07 |
| Axial length (mm) | 24.82 (0.11) [24.60, 25.03] | 24.54 (0.18) [24.10, 24.97] | 0.32 |
| Age at myopia onset (years) | 9.34 (0.22) | 9.43 (0.61) | 0.89 |
|  | [8.89, 9.79] | [7.93, 10.93] |  |
| Parents with myopia, No. (%) | |  | 0.40 |
| 0 | 16 (36.4) | 2 (28.6) |  |
| 1 | 14 (31.8) | 4 (57.1) |  |
| 2 | 14 (31.8) | 1 (14.3) |  |
| SER changes in 3 years (D) | −0.91 (0.11) [−1.14, −0.68] | −1.45 (0.27) [−2.11, −0.79] | 0.17 |
| AL elongation in 3 years, (mm) | 0.47 (0.05) [0.37, 0.56] | 0.65 (0.14) [0.31, 1.00] | 0.09 |

HAL= spectacle lenses with highly aspherical lenslets; SER = spherical equivalent refraction; D = diopters

**Figure S1. Flowchart of the study, showing participant numbers over 5 years.**

HAL, spectacle lenses with highly aspherical lenslets; SAL, spectacle lenses with slightly aspherical lenslets; SVL, single-vision spectacle lenses; HAL2, children who had worn SAL in the previous 2-year study and had switched to wearing HAL in the 3^rd^ year; HAL3, children who had worn SVL in the previous 2-year study and had switched to wearing HAL in the 3^rd^ year; SVL2, a new single-vision spectacle lenses group as a control in 3^rd^ year; ESVL, extrapolated single-vision spectacle lenses; SER, spherical equivalent refraction; AL, axial length.


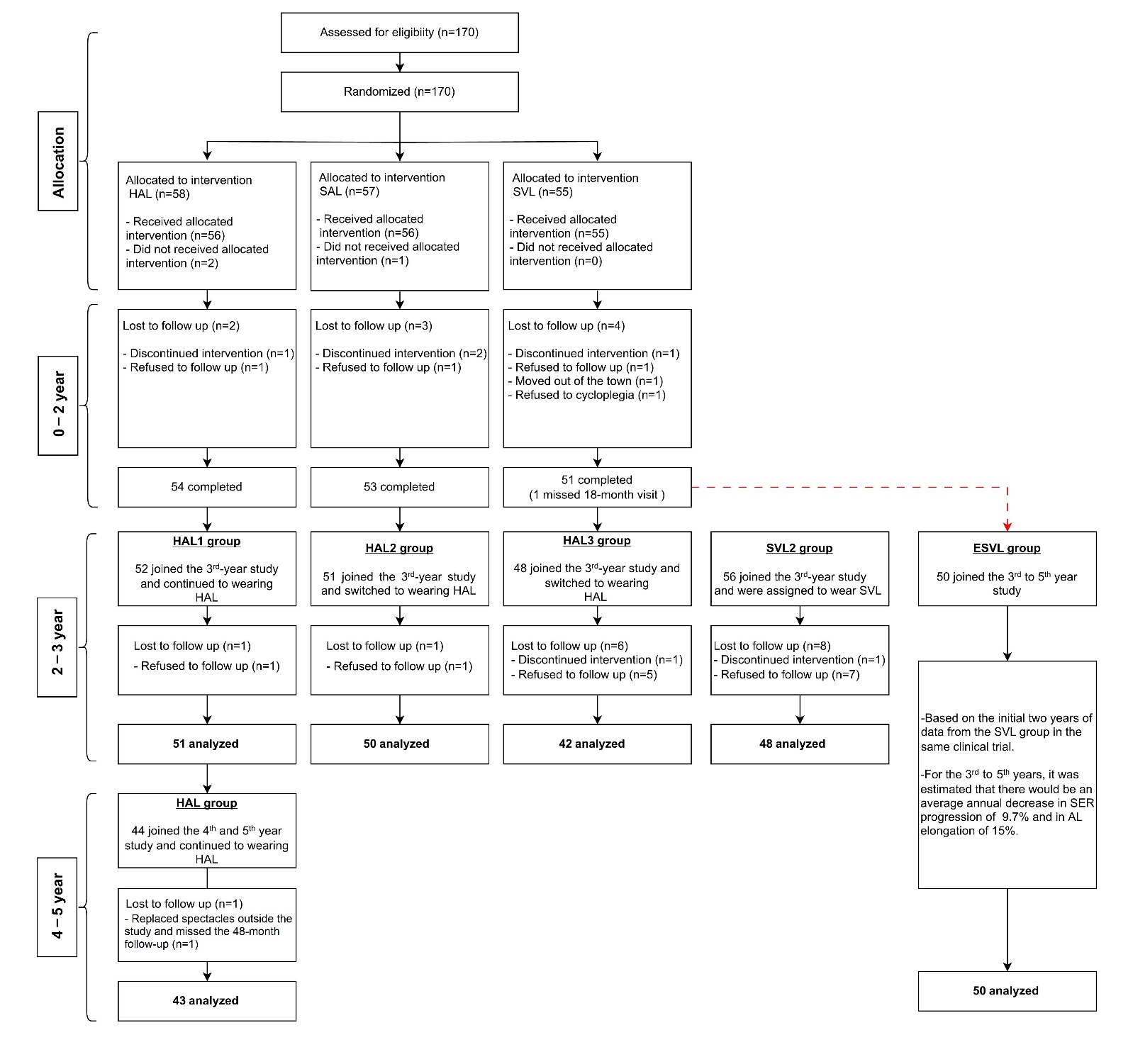

Supplement: Supplementary file 1 — Additional file 1. [file 40662_2025_427_MOESM1_ESM.docx]
